# Sickle cell trait (HbAS) and stunting in children below two years of age in an area of high malaria transmission

**DOI:** 10.1186/1475-2875-8-16

**Published:** 2009-01-16

**Authors:** Benno Kreuels, Stephan Ehrhardt, Christina Kreuzberg, Samuel Adjei, Robin Kobbe, Gerd D Burchard, Christa Ehmen, Matilda Ayim, Ohene Adjei, Jürgen May

**Affiliations:** 1 Section for Tropical Medicine, Medical Department I, University Medical Centre Hamburg-Eppendorf, Hamburg, Germany; 2 Infectious Disease Epidemiology, Bernhard Nocht Institute for Tropical Medicine, Hamburg, Germany; 3 Clinical Research, Bernhard Nocht Institute for Tropical Medicine, Hamburg, Germany; 4 Ministry of Health/Ghana Health Service District Health Directorate, Agona, Ashanti Region, Ghana; 5 Department of Paediatrics, University Medical Centre Hamburg-Eppendorf, Hamburg, Germany; 6 Department of Molecular Medicine, Bernhard Nocht Institute for Tropical Medicine, Hamburg, Germany; 7 Kumasi Centre for Collaborative Research in Tropical Medicine, Kumasi, Ghana

## Abstract

**Background:**

While the protective effects of sickle cell trait (HbAS) against severe malaria and the resulting survival advantage are well known, the impact on the physical development in young children remains unclear. This study was aimed to investigate the relationship between HbS carriage and stunting in children below two years of age in a cohort from the Ashanti Region, Ghana.

**Methods:**

1,070 children were recruited at three months of age and followed-up for 21 months with anthropometric measurements performed every three months. Incidence rate ratios with 95% confidence intervals were calculated by Poisson regression to estimate the association of β-globin genotypes with the number of malaria episodes. Odds ratios (OR) were calculated for the association between the occurrence of β-globin genotypes and/or malaria episodes and stunting. The age-dependent between-group and within-group effects for the β-globin genotypes were assessed by population-averaged models estimated by generalized estimation equation with autoregressive correlation structure.

**Results:**

Analyses showed a significantly lower age-dependent risk of stunting (OR 0.56; 95% CI 0.33–0.96) in carriers of the HbAS genotype (n = 102) in comparison to those with HbAA (n = 692). This effect was restricted to children who experienced malaria episodes during the observation period suggesting that the beneficial effect of the β-globin HbS variant on the incidence of stunting is closely linked to its protection from mild malaria episodes.

**Conclusion:**

The lower risk of chronic malnutrition in early childhood, mediated by protection against mild malaria episodes, may contribute to the survival advantage of HbAS carriers in areas of high malaria transmission.

## Background

There is much epidemiological evidence that sickle cell trait decreases the risk for all manifestations of severe malaria and that this protective effect has caused a balanced polymorphism of the sickle cell mutation (HbS) in malaria endemic regions [1]. In addition to protection against severe malaria, HbS has also been shown to exhibit some level of protection against mild malaria [2]. The importance of this observation for the survival advantage of individuals with HbAS remains unclear [3]. Repeated malaria episodes in early childhood can impair a child's development and induce malnutrition [4-6]. Stunting (length/height-for-age *z*-score < -2) in early childhood, as an indicator of chronic malnutrition, is a common condition in African children and one of the main determinants of childhood morbidity and mortality [7]. It has also been demonstrated, that stunting is related to poor cognitive performance later on in life and has a negative effect on adult health and human capital [8,9].

So far, most studies on the effect of HbAS on physical development were performed in areas without malaria transmission. In the majority of these studies no significant differences between individuals with the HbAA and HbAS genotype were found [10,11], while some investigators found a slight impairment of development in HbAS carriers [12]. Results of the only study performed in an area of low malaria endemicity also showed no protection of HbAS carriers against stunting [4]. The hypothesis of the present study was, that in areas with high malaria transmission, HbAS may, through protection against malaria, also confer protection against stunting in young children.

## Methods

In the frame of a trial on Intermittent Preventive Treatment in infants (IPTi), conducted from January 2003 to September 2005, a cohort of 1,070 children from a rural district in the Ashanti Region of Ghana was recruited at the age of three months and followed-up monthly until two years of age. On each visit a clinical examination was performed, a standardized medical history taken and parasite density measured. The length of each child was assessed at recruitment and subsequently in three-monthly intervals, with measurements performed on the same standardized length board for all children. Details on the study area, study group and procedures have been published elsewhere [13,14]. A malaria episode was defined as body temperature > 38°C or a history of fever in the preceding 48 h and a parasite density of more than 500 parasites/μl. Stunting was defined as a length/height-for-age *z*-score of less than -2 according to WHO (moderate and severe stunting combined) [15]. DNA preparation and genotyping of the β-globin gene were performed according to standardized protocols as described elsewhere [16].

Statistical analyses were performed with STATA (version 10.0, StataCorp, USA). Only children with HbAA or HbAS genotype and with at least six completed anthropometric measurements were considered for the analyses. Missing values for anthropometric data between two existing follow-up visits were interpolated by calculating the mean of the two flanking visits (in total 5883 anthropometric measurements and 342 interpolated values). P values below 0.05 were considered significant. Incidence rate ratios (IRR) with 95% confidence intervals (CI) were calculated by Poisson regression to estimate the association of β-globin genotypes with the number of malaria episodes. Odds ratios (OR) were calculated for the association between the occurrence of β-globin genotypes and/or malaria episodes and stunting. The age-dependent between-group and within-group effects for the β-globin genotypes were assessed by population-averaged models estimated by generalized estimation equation (GEE) with autoregressive correlation structure of order 5 and assumption of binomial variable distribution. Autoregression was used to account for intra-individual age-dependency of anthropometric measurements and the most appropriate autoregression order was assessed by correlation matrices. Resulting coefficients were log-transformed and given as ORs with CI to estimate the risks for stunting at three-monthly cross-sectional time-series.

## Results

**Figure 1:**
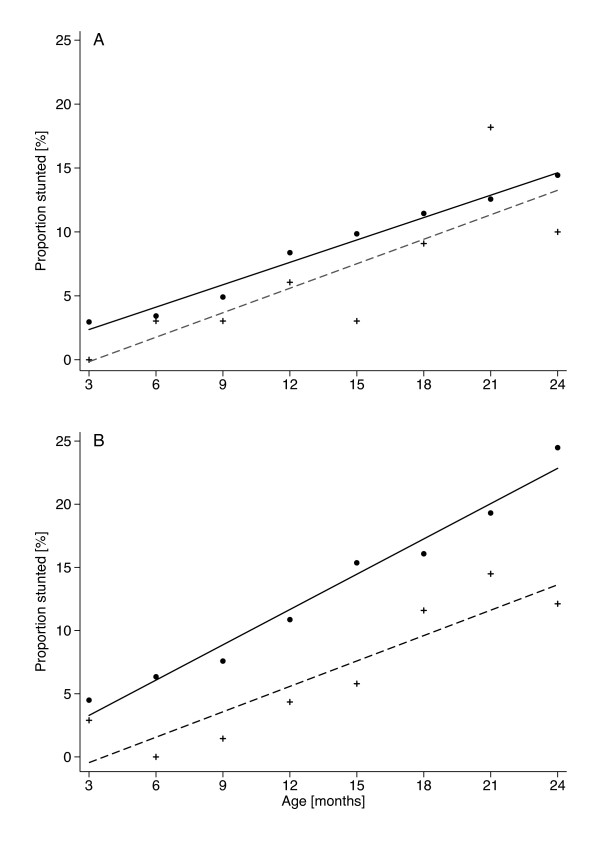
**Relationship between stunting and age in children with and without malaria episodes**. Proportion of children stunted with increasing age stratified for b-globin genotypes HbAA (dots and solid line) and HbAS (crosses and dashed line). GEE analyses with adjustment for within-group, between-group effects and age-dependency were performed with autoregressive correlation structure of order 5 and assumption of binomial variable distribution. A, children without malaria episodes; no significant protective effect of HbAS (OR 0.84; CI 0.33–2.15; p = 0.71). B, children with at least one episode of malaria; 51% lower risk of stunting (OR 0.49; CI 0.25–0.93; p = 0.03).

Out of the 1,070 children recruited, 794 children were included in the analyses as they were carriers of the HbAA or HbAS genotype and had completed at least six anthropometric measurements. A total of 692 children were HbAA homozygotes while 102 children were carriers of HbAS (Table 1). At recruitment (three months of age) 4% of all children in the study group were stunted, while at the end of the follow-up period (two years of age) this proportion had increased to 20%. In children who were not stunted at recruitment, HbAS carriers had a 58% decreased risk of being stunted at two years of age in comparison to HbAA carriers (OR 0.42; CI 0.20–0.86; p = 0.016). GEE analyses with autoregression showed that children with the HbAS genotype had a 44% lower age-dependent risk of stunting during the first two years of life (OR 0.56; CI 0.33–0.96; p = 0.034). A sub-group analysis showed that the effect was restricted to children with at least one episode of malaria (OR 0.49; CI 0.25–0.93; p = 0.03). In contrast, children without any malaria episodes HbAS carriers were not protected against stunting (OR 0.84; CI 0.33–2.15; p = 0.71) (Figure 1).

**Table 1:** Characteristics of study participants at recruitment

	β-globin genotype^a^	
	
	HbAA (n = 692)	HbAS (n = 102)
Mean length/height-for-age z-score (sd)^b^	0.31 (± 1.22)	0.33 (± 1.11)
		
Clinical malaria (%)		
No	678 (98.1)	101 (98.0)
Yes	13 (1.9)	1 (1.0)
		
Breastfeeding (%)		
Exclusively	594 (86.0)	94 (92.2)
Non-exclusively	97 (14.0)	8 (7.8)
		
Mean birth-weight (sd), g^c^	2996 (± 471)	3085 (± 366)
		
Ethnic group (%)		
Ashanti	615 (89.4)	88 (86.3)
Northerner	73 (10.6)	14 (13.7)
		
Mother's literacy (%)		
Literate	620 (90.6)	91 (91.0)
Illiterate	64 (9.4)	9 (9.0)
		
Mosquito protection (%)		
None	290 (44.1)	54 (57.4)
Bed nets^d^	234 (35.6)	29 (30.9)
Screens	134 (20.4)	11 (11.7)
		
Financial situation (%)		
Good	458 (70.5)	66 (70.2)
Poor	192 (29.5)	28 (29.8)
		
α^+^-thalassemia (%)		
wild type	506 (74.3)	76 (75.2)
heterozygotes	159 (23.4)	22 (21.8)
homozygote deletion	16 (2.3)	3 (3.0)
		
Study arm (%)		
IPTi	345 (49.9)	50 (49.0)
Placebo	347 (50.1)	52 (51.0)

sd, standard deviation.

^a ^missing values possible due to incomplete or unvalidated parameters.

^b ^n = 794.

^c ^n = 547.

^d ^treated and untreated bed nets.

In addition to being protected against stunting, HbAS carriers in this study were also protected against mild malaria episodes (IRR 0.82; CI 0.69–0.96; p = 0.017). In order to analyze a direct effect of malaria episodes on stunting, the ORs for the risk of stunting at the end of the study period were calculated only for children who were not stunted at recruitment. Children with two or more episodes of malaria during the study period had an increased risk of becoming stunted at two years of age compared to children with zero or one episode (OR 1.71; CI 1.17–2.51; p = 0.006). Other factors with reported influence on stunting (e.g. breastfeeding, birth-weight, ethnic group, mother's literacy, mosquito nets/screens and financial status) and other genetic hemoglobin disorders (α^+^-thalassemia) were tested for effect modification but no influence on the effect of HbAS and malaria on stunting was detected. There was also no effect modification by the study-arm (IPTi vs. placebo).

## Discussion

While the protective effect of HbAS on the risk of severe malaria is well known [2,3], protection of HbAS carriers against mild malaria episodes and a subsequent effect on general child health remains unclear [17]. This study was aimed to assess the effect of HbAS on mild malaria and to identify a possible effect on childhood development. As expected, HbAS carriers were significantly protected against mild malaria episodes in the study presented here. However, when compared to HbAA carriers, HbAS carriers also demonstrated a lower risk of stunting during the first two years of life. Such a direct association between sickle cell trait and stunting has not been reported before. Since malaria itself has been positively associated with stunting in the past [4-6], it seems possible that a protective effect of the sickle cell trait against malaria could explain the decreased risk of HbAS carriers for stunting. The fact that malaria was also found to increase the risk of being stunted at two years of age in this study further supports this hypothesis. Notably, previous studies on the relationship between HbAS and early childhood development that found no protection of HbAS against stunting were all conducted in areas without or with only low malaria transmission [4,10-12]. A sub-group analysis of children without any malaria during the study period showed no protective effect of HbAS carriers against stunting. This also indicates that an effect of HbAS on stunting may only be exhibited in populations highly exposed to malaria and may explain why this effect was not detected in earlier studies. A further reason why previous studies failed to find an association might be the age range under study. The protective effect of HbAS is greatest at young age and becomes less distinct later in life [3]. While this study focuses on children between three months and two years of age, other studies also included older children. Due to the intensive follow-up scheme and medical interventions in case of illness as well as regular substitution of iron, folic acid, vitamin A and zinc, fewer children than expected were stunted. Substitution of micronutrients and vitamins did not differ between the two groups and is therefore unlikely to have confounded the results. The lower incidence of stunting however may have lead to an indifferential error and an underestimation of the protective effect of HbAS against stunting. A further limitation could be a follow-up bias due to the restriction of the analysis to children who were available for six anthropometric measurements. This bias, however, might be minimal since only 19 children were lost to follow-up due to early death.

## Conclusion

Young children with sickle cell trait (HbAS) have a lower risk of stunting in an area with high malaria transmission, most likely mediated through protection against mild malaria. Since stunting in early years is known to be a strong risk factor for morbidity and mortality and considering the high incidence of mild malaria, it seems probable that protection against stunting contributes essentially to the survival advantage of HbAS carriers.

## Abbreviations

IPTi: Intermittent Preventive Treatment in infants; HbS: Sickle cell mutation; GEE: Generalized Estimation Equation; OR: Odds Ratio; IRR: Incidence Rate Ratio; CI: 95% Confidence Interval.

## Competing interests

The authors declare that they have no competing interests.

## Authors' contributions

BK collected data, performed statistical analyses and wrote the first draft of the manuscript. SE contributed to the writing of the manuscript. CK, RK and SA organized and supervised the study in Ghana and collected data. GDB critically reviewed the manuscript. CE and MA performed the laboratory work. OA was principal investigator of the study and JM designed and implemented the study, performed statistical analyses and was involved in the writing of the manuscript. All authors contributed significantly to the final version of the manuscript.
